# Development of a methodology for the volume estimation of the prefrontal cortical subfields in very pre-term infants using magnetic resonance imaging and stereology

**DOI:** 10.2478/abm-2025-0023

**Published:** 2025-09-02

**Authors:** Faten Aldhafeeri

**Affiliations:** Department of Health Information Management and Technology, University of Hafr Al-Batin College of Applied Medical Sciences, Hafar Al Batin 39953, Saudi Arabia

**Keywords:** grey matter, magnetic resonance imaging, PFC, stereology, volume estimation

## Abstract

**Background:**

The prefrontal cortex (PFC) is vital for cognitive and emotional functions and is vulnerable to disruptions in preterm infants. Reliable volume estimation methods are needed to study its development.

**Objective:**

To develop and validate a novel method for estimating the volume of PFC subfields in very preterm infants using magnetic resonance imaging (MRI) combined with stereological techniques. The method was designed to achieve a coefficient of error (CE) below 5%.

**Methods:**

Five preterm infants born before 28 weeks of gestation were scanned using a 1.5-Tesla MRI scanner. The points of intersection between the grid and structure boundaries, in addition to the points in each slice, were counted using in-house software (Easy Measure).

**Results:**

The shape coefficient for each subfield of the prefrontal cortex was calculated, which yielded coefficients of 4.5, 6.1, 6.4, and 6.5 for dorsolateral, dorsomedial, orbitolateral, and orbitomedial PFC regions, respectively. For the dorsolateral prefrontal cortex, a grid size of 4 × 4 pixels and a 0.2 cm slice gap for the dorsomedial prefrontal cortex (DMPFC), a grid size of 5 × 5 pixels and a 0.1 cm slice gap for the orbitolateral PFC, a grid size of 5 × 5 pixels and a 0.3 cm slice gap, and a grid size of 5 × 5 pixels and 0.1 cm slice gap for the DMPFC resulted in <5% CE.

**Conclusion:**

This methodology offers new insights into the neurodevelopmental effects of preterm birth and has potential applications in the early detection of neurodevelopmental disorders. Its precision, reliability, and non-invasive nature make it suitable for longitudinal studies and contribute to neonatal neuroimaging and neurodevelopmental research.

The prefrontal cortex (PFC) is a brain area involved in various cognitive activities and developmental processes. It is considered the foundation for higher cognitive processes [1]. The development of the PFC is a multifaceted process that occurs gradually over an extended period and encompasses a variety of factors, including neural innervation, cognitive control, and environmental influences. The PFC has been the subject of numerous studies to examine its developmental changes during adolescence. These studies have revealed that the PFC undergoes substantial maturation during this period, characterized by the integration and refinement of various brain functions that support cognitive development [2, 3]. This region of the brain plays an important role in higher-order cognitive functions, such as decision-making, impulse control, and working memory [2]. The maturation of the PFC is closely associated with the development of these cognitive abilities during adolescence [4]. The maturation of the PFC is not limited to a single localized area, but involves a widely distributed brain network that is responsible for coordinating and integrating information from different brain regions, thus enabling efficient cognitive processing. The incorporation and maturation of these networks contribute to the overall cognitive development observed during adolescence [5].

The PFC is important in pediatric development because it regulates cognitive control, emotional regulation, and memory. It serves as a bridge between domain-specific working memory representations and mental activity [6]. During early childhood, it enables complex behavior and cognitive shifting [7, 8]. Moreover, it is involved in regulating emotion, as lower activation of the ventrolateral and dorsolateral PFC during negative emotion downregulation has been reported across clinical groups [9]. Finally, it has been implicated in the regulation of stress responses, disruption of sleep patterns, and manifestation of depressive symptoms, thereby underscoring its significance in the realm of psychological well-being [10].

Stereology is a mathematically unbiased method used to extract quantifiable information of a 3D object from two-dimensional (2D) sections. It uses mathematical tools, geometry, statistics, and most importantly, common sense [11]. For biological structures, stereology is used to obtain estimates of the geometrical characteristics of a structure, including length, cell number, surface, and volume. Different stereological methods have been developed to estimate brain volume. Recent advances in imaging, such as magnetic resonance imaging (MRI) and computed tomography, have enabled researchers to estimate brain volume non-invasively [12]. The Cavalieri principle underlies most stereological studies in conjunction with point counting, which states that the volume of an arbitrary object can be estimated from the cross-sectional areas of parallel sections separated by a known distance [13]. The Cavalieri method is efficient for volume estimation because it is mathematically unbiased, provided that the first slice is randomly selected from within the first gap and the structure is extensively sectioned. Furthermore, it is not affected by the object's shape or the orientation of the slices [14, 15].

Estimating the PFC contributes to an understanding of various aspects of brain functionality and its implications on health and neurological disease. Numerous studies have used advanced neuroimaging techniques and stereology to examine the complex relationship between PFC volume and various aspects of cognitive function, mental health, and developmental changes. For example, studies have demonstrated that the volume of the PFC correlates with executive functions in individuals without neurological disorders. This highlights the importance of using quantitative methods to elucidate the connections between PFC volume and cognitive abilities, as evidenced by the work of Yuan and Raz [16]. Furthermore, numerous studies have been conducted to determine the intricate connection between the PFC volume and the size of an individual's social network. These studies have provided insight into the potential implications of estimating PFC volume to understand the variations observed in social behavior among individuals [17].

Optimization of stereological methods allows for volume estimation to be made at higher precision levels (i.e., the estimated brain volume will be closer to the actual brain volume and also more efficiently computed). The coefficient of error (CE) is a representation of the reproducibility of the results and is considered an assessment tool to evaluate the sampling method through which the volume is estimated. Furthermore, the mean CE assesses the contribution of the group mean variance to the stereological scheme. The CE of the estimated volume may be calculated from the point counts on a group of slices [18]. Recent studies indicate the contribution of variability between sections and the variability that results from point counting within sections when predicting the true square CE of *V͂*, as shown in the following equation:

(1) $${\text{CE}}^{2}(\tilde{V})={\text{CE}}^{2}(\tilde{V})+{\text{CE}}_{PC}^{2}(\tilde{V})$$

where CE^2^ (*V̂*) is the true contribution of the error resulting from variability among sections, and ${\text{CE}}^{2}(\hat{V})$ is the true mean variability due to point counting within sections [12]. The contribution of the sections and point counting is estimated as follows [19]:

(2) $${\text{CE}}_{PC}^{2}(\tilde{V})$$

The dimensionless shape coefficient $\begin{array}{l} {\text{CE}}^{2}(\hat{V})=\alpha (q)\cdot (3(C_{0}-\hat{\upsilon })-4C_{1}+C_{2}).\\ {\left(\Sigma Pi\right)}^{-2},\;ce_{PC}^{2}(\tilde{V})=\hat{\upsilon }\cdot {\left(\Sigma Pi\right)}^{-2} \end{array}$ provides information on the average shape of the sections and indicates the shape complexity. *B̄* is an estimator of the mean boundary length, where *Ā* estimates the mean area of the sections. The shape coefficient for the individual structures is calculated as follows:

(3) $$\frac{\bar{B}}{\sqrt{\bar{A}}}/\sqrt{\bar{A}}$$

where *I* is the number of intersections between the grid and the shape boundaries, *P* is the number of points counted, *l* is the length associated with one point, and *m* is the number of slices. CE does not represent the contribution of biological variances, as it primarily evaluates the sampling scheme by which the volume of an object is estimated [20].

In the present study, we applied the Cavalieri method of stereology in combination with point counting to estimate the volume of four subfields of the PFC: the lateral and medial orbital PFC volume and the lateral and medial dorsal PFC volume [21]. The shape coefficient for each PFC subfield was evaluated to optimize the sampling strategy for the PFC volume estimation of the brains of preterm infants with a predicted coefficient error <5%. Moreover, the stereological parameters for the PFC volume estimation of preterm infants were standardized, which can be applied to future studies.

## Methods

The present study was conducted in accordance with the ethical standards of the University of Hafr Albatin, Saudi Arabia, and with the 1964 Helsinki Declaration and its later amendments. Ethical approval was obtained from the Directorate of Health Affairs. Research Ethics Committee (COA no: HPO/23051876).

Written informed consent was obtained from the parents or legal guardians of all infants prior to their inclusion in the study. Patients' data were anonymized to ensure confidentiality and privacy throughout the research process.

Five infants were recruited of a gestational age under 28 weeks at birth and were scanned in a 1.5T MRI scanner (Philips Medical Systems) immediately following feeding (to avoid sedation). T1-weighted Turbo Spin Echo (TSE) sequence images were acquired by applying the following parameters: TR = 3023 ms, TE = 150 ms, slice thickness = 3 mm, in-plane isotropic resolution of 0.7 mm, matrix size 256 mm × 256 mm, and scan time = 3 min. A phased array sensitivity encoding (SENSE) head coil will be used to facilitate the production of high-quality MR images with a high signal-to-noise ratio. Although 3D MRI sequences are commonly preferred for volumetric brain analyses because of their isotropic resolution, the current study employed a 2D T1-weighted TSE sequence. This decision was based on practical considerations related to scanner availability, neonatal imaging protocols, and the need to minimize the scan duration for very preterm infants. The application of the Cavalieri method in conjunction with point counting enables unbiased and accurate volume estimation using 2D datasets, provided that systematic random sampling and appropriate sectioning protocols are followed. Previous studies have indicated the validity of stereological techniques, such as the Cavalieri principle, in estimating brain volume from 2D MR images, even in the absence of isotropic voxels. The imaging parameters (slice thickness of 3 mm, in-plane resolution of 0.7 mm, and standardized anatomical landmarks) were carefully selected to support the precision of the stereo-logical assessment. Clinical data were extracted from the neonatal medical records to provide further context for the cohort. All 5 infants were born at <28 weeks of gestation (mean gestational age: 26.2 ± 0.8 weeks; mean birth weight: 890 ± 120 g). The underlying reasons for preterm delivery included preeclampsia (n = 2), premature rupture of membranes (PROM) (n = 1), and spontaneous preterm labor with no identifiable cause (n = 2). The relevant comorbidities included broncho-pulmonary dysplasia (n = 2) and neonatal jaundice requiring phototherapy (n = 1). None of the infants had major congenital anomalies or required surgical intervention during the neonatal period (**Table 1**).

**Table 1.** Clinical characteristics of preterm infants

**Infant ID**	**Gestational age (weeks)**	**Birth weight (g)**	**Reason for preterm delivery**	**Comorbidities**
01	25 + 6	870	Preeclampsia	Bronchopulmonary dysplasia
02	26 + 2	920	PROM	None
03	27 + 1	980	Spontaneous preterm labor	None
04	26 + 0	860	Spontaneous preterm labor	Hyperbilirubinemia
05	25 + 5	910	Preeclampsia	Bronchopulmonary dysplasia

PROM, premature rupture of membranes.

### Parcellation of the PFC subcortical fields

The division of the PFC was based on the method described by Howard et al. [21]. For each cerebral hemisphere, the PFC was divided into the following 4 regions: dorsolateral (DL), dorsomedial (DM), orbitolateral (OL), and orbitomedial (OM). To prevent interindividual and interhemispheric variations, such as discontinuities appearing in gyral and sulcal landmarks within the frontal lobe, the subcortical and midline structures were used as landmarks. The orbital and dorsal subfields were divided along the commissural plane, which was demarcated as a horizontal line in all slices. For the medial and lateral demarcation, the first axial slice superior to the olfactory sulcus was used to separate the medial aspect for the dorsal and orbital subfields from the lateral one. Slice numbers indicating the location of the medial and lateral boundaries in the sagittal plane were noted for the right hemisphere, because we are only estimating the volume of the right PFC subfields. This lateralization was selected to ensure methodological consistency and minimize the analysis time. The right hemisphere was selected based on existing evidence, which suggests a greater susceptibility of the right PFC to early life stress and injury [22, 23]. The posterior boundary of the dorsal regions (lateral and medial) was indicated by the genu of the corpus callosum observed at the sagittal midline. On the other hand, the posterior boundary of both orbital regions, medial and lateral, was readily demarcated, as the natural anatomical borders for this region are identifiable [21].

### Volume estimation and evaluation of the shape coefficient

**Figure 1.**
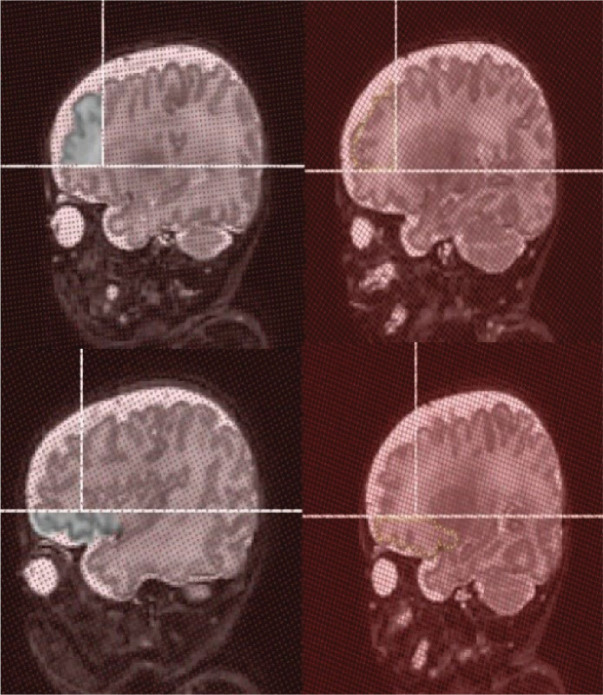
Cross-sectional area point counting (points removed) for the DL PFC and OL PFC (the upper and lower left frames, respectively). Boundary length estimation for the DL PFC and OL PFC (the upper and lower right frames, respectively). The stereological grids appear as red crosses in the cross-sectional area point counting, and as red lines in the boundary length estimation. The yellow crosses indicated demonstrate the intersections between the structure boundaries and the grid. DL, dorsolateral; OL, orbitolateral; PFC, prefrontal cortex.

Demarcation of the anatomical landmarks, reorientation of the slices, and alignment were performed using the Brain Voyager software. The point counting technique in stereology is a statistical method for volume estimation of an object [24]. The CE represents the stereological decision of stereological estimation and is also considered a statistical expression for the size of the SEM of the repeated volume estimates [20]. Points of intersection between the grid and shape boundaries were calculated using Easy Measure software with a minimum grid size of 2 × 2 pixels and a 0.1-cm slice gap. Moreover, the total number of counted points in each slice was calculated and exported to an Excel file. The boundary length estimation was determined by calculating the total number of intersections between the structure and the grid lines. Subsequently, the exported Excel file data reflected the total number of intersections. The cross-sectional area was estimated by calculating the total points in each slice, while considering only white and gray matter (**Figure 1**). The total number of intersections and points counted on the cross-sectional area was used in Eq. (3) to calculate the shape coefficient.

### Inter- and intra-rater reliability

The inter- and intra-rater reliability of the morphometric measures of the four PFC subfields was analyzed using intraclass correlation coefficients. For inter-rater reliability, a second independent rater, blind to the purpose of the study, performed morphometric measurements for all subjects. Intra-rater reproducibility was determined by repeating the PFC subfields measurements of all subjects with at least 4 weeks between the two measurements.

## Results

Inter- and intra- rater reliability measures ranged between 0.86–0.97 and 0.94–0.97, respectively. The shape coefficient (calculated using Eq. (3)) for the DL, DM, OL, and OM is shown in **Table 2**.

**Table 2.** The calculated shape coefficient for PF subcortical fields

**Region**	**Shape coefficient**
DL	4.5
DM	6.1
OL	6.4
OM	6.5

DL, dorsolateral; DM, dorsomedial; OL, orbitolateral; OM, orbitomedial.

In addition, the CE was calculated by adjusting the grid sizes and slice intervals for each prefrontal subcortical region in the right hemisphere (**Tables 3–6**).

**Table 3.** The calculated CE for the estimated volume of the DLPFC subfield

**Grid size (pixels)**		**Slice gap (cm)**		
		**0.1**	**0.2**	**0.3**
2	CE (%)	2.176	4.145	6.46
	V (mm^3^)	8.428	8.152	8.316
3	CE (%)	2.231	4.254	6.546
	V (mm^3^)	8.424	8.154	8.343
4	CE (%)	2.348	4.382	6.717
	V (mm^3^)	8.480	8.192	8.352
5	CE (%)	2.729	4.899	7.14
	V (mm^3^)	8.175	7.900	8.250

CE, coefficient of error; DLPFC, dorsolateral prefrontal cortex.

**Table 4.** The calculated CE for the estimated volume of the DMPFC subfield

**Grid size (pixels)**		**Slice gap (cm)**		
		**0.1**	**0.2**	**0.3**
2	CE (%)	4.05	8.468	12.161
	V (mm^3^)	7.276	8.016	8.496
3	CE (%)	4.097	8.499	12.264
	V (mm^3^)	7.290	8.028	8.505
4	CE (%)	4.201	8.627	12.384
	V (mm^3^)	7.280	8.032	8.496
5	CE (%)	4.282	8.698	12.249
	V (mm^3^)	7.325	8.050	8.550

CE, coefficient of error; DMPFC, dorsomedial prefrontal cortex.

**Table 5.** The calculated CE for the estimated volume of the OLPFC subfield

**Grid size (pixels)**		**Slice gap (cm)**		
		**0.1**	**0.2**	**0.3**
2	CE (%)	1.992	4.256	6.078
	V (mm^3^)	8.592	8.224	8.208
3	CE (%)	2.119	1.136	1.402
	V (mm^3^)	8.577	8.190	8.208
4	CE (%)	1.188	1.734	2.15
	V (mm^3^)	8.640	8.288	8.256
5	CE (%)	1.659	2.42	2.986
	V (mm^3^)	8.650	8.300	8.325
6	CE (%)	2.183	3.188	3.928
	V (mm^3^)	8.640	8.280	8.316
7	CE (%)	2.732	3.999	4.922
	V (mm^3^)	8.722	8.330	8.379

CE, coefficient of error; OLPFC, orbitolateral prefrontal cortex.

**Table 6.** The calculated CE for the estimated volume of the OMPFC subfield

**Grid size (pixels)**		**Slice gap (cm)**		
		**0.1**	**0.2**	**0.3**
2	CE (%)	3.227	6.36	7.946
	V (mm^3^)	6.544	7.064	7.464
3	CE (%)	3.449	4.944	8.047
	V (mm^3^)	6.831	7.614	7.479
4	CE (%)	3.393	6.493	8.071
	V (mm^3^)	6.576	7.072	7.488
5	CE (%)	3.688	6.841	8.567
	V (mm^3^)	6.550	7.100	7.500
6	CE (%)	3.866	6.899	8.639
	V (mm^3^)	6.516	7.056	7.344
7	CE (%)	4.243	7.38	8.999
	V (mm^3^)	6.468	6.958	7.350

CE, coefficient of error; OMPFC, orbitomedial prefrontal cortex.

The increase in grid size would result in a reduction in the number of points to be counted, and therefore, decrease the time required to perform stereology, thus increasing the efficiency of the sampling strategy. Furthermore, increasing the grid size only has negligible effects on the CE compared with increasing the slice gap. A sampling strategy was successfully developed that provides sufficient precision to estimate the PFC subfields in preterm infants, in addition to determining stereological parameters that may be applied in future studies. The optimized stereological method provides quick and mathematically unbiased PFC volume estimations of very preterm infants. **Table 7** demonstrates the recommended stereological parameters identified to estimate the PFC volume for preterm infants under 28 weeks of gestation.

**Table 7.** Recommended stereological parameters

**PFC subfield**	**Grid size (pixels)**	**Slice gap (cm)**	**Shape coefficient √B/√√A**	**CE (%)**
DL	4 × 4	0.2	4.5	4.38
DM	5 × 5	0.1	6.1	4.28
OL	5 × 5	0.3	6.4	3.92
OM	5 × 5	0.1	6.5	3.86

CE, coefficient of error; DL, dorsolateral; DM, dorsomedial; OL, orbitolateral; OM, orbitomedial; PFC, prefrontal cortex.

## Discussion

Quantitative MRI was performed using three-dimensional software based on the manual or semiautomatic segmentation of anatomical structures. The accuracy and validity of stereology as a quantitative method for volume estimation of neuroanatomical structures are well documented [24,25,26]. In the present study, a stereological method was established to provide highly precise (CE <5%) estimated volumes of PF subcortical fields for preterm infants born under 28 weeks of gestation and to determine stereological parameters for future use. To our knowledge, this is the first study describing a sampling strategy for estimating the right PFC volumes of very preterm infants. The validity of the point counting technique combined with MR images has been documented [14].

The application of stereological techniques, such as the Cavalieri method, point counting, and physical dissection, has made it possible to precisely estimate the volume of the PFC subfields, encompassing the dorsal and orbital regions. This method was shown to be of significant use in examining the ramifications of trauma and stress on the volume of the PFC, as demonstrated by studies delving into the correlation between diminished PFC volume and heightened secretion of cortisol in individuals who have experienced trauma during their developmental years. Moreover, stereological methods have been used to assess the overall quantity of neurons, glial cells, and the compactness of astrocytes in the PFC, thereby providing insight into this particular area of the brain [22, 27].

Numerous techniques are available for estimating the volume of various brain compartments from MR images. Manual techniques are user-dependent and require the user to delineate brain regions based on either anatomical or functional landmarks. Two common manual techniques were used to estimate the volume of distinct brain regions: stereology in conjunction with point counting and tracing methods. Both techniques achieved good efficiency and precision. On the other hand, the automated techniques are user-independent.

Many studies have developed methodologies for PFC parcellation to perform stereology. Gur et al. [28] used a software package that allows the manual outlines of the anatomical landmarks to be traced across several slices. Then, the slice areas were summed to yield volume estimates. Others have developed a computer-assisted algorithm that automatically identifies the anatomical landmarks [29, 30]. The combination of manual and automated techniques has been used in several studies. For example, Ranta et al. [31] performed a parcellation of the frontal lobe using a software package that relies on sulcal/gyral landmarks. The landmarks were selected for use in the delamination of the functionally relevant frontal regions. Similar to this methodology, Ranta et al. [32] manually isolated the frontal lobe from the rest of the brain and subdivided it into 11 regions. The current methodology relies on region-drawing techniques that have shown high reliability and reproducibility [28]. It expands upon previous techniques by focusing on divisions that anatomically outline distinct regions of the PFC [33, 34]. The easy implementation of the Cavalieri method, in addition to its efficiency, has made it common in many studies examining brain volume [34, 35].

The present study aimed to optimize the stereological parameters for preterm infants, because there are no current studies that propose parameters to estimate the PFC subfields. The estimation of PFC volume in infants holds significant value because of its pivotal involvement in the early stages of brain development and its consequential impact on cognitive, emotional, and social function [36]. The PFC experiences substantial maturation during the early stages of infancy, and its volume is linked to a range of developmental outcomes and neurological conditions. Thus, the precise assessment of PFC volume in infants is important to understand the trajectory of neurodevelopment and identify variables that may influence cognitive and emotional health [37,38,39]. Moreover, studying the volume of the PFC in infants may provide insight into the effect of prenatal exposures, such as perfluorinated compounds (PFCs), on brain development. A study showed that exposure to PFCs in infants may have consequences for brain development. Therefore, accurately estimating PFC volume is important to understand the potential effects of environmental contaminants on early brain growth and function [40].

Optimization of a stereological method can be done by either altering the testing grid size, slice gaping, or both. Salomianka and West [20] demonstrated that when the sections were closer to one another, the CE estimates decreased. There was an effect of both grid size and slice gap on the CE when estimating the DLPFC subfield. However, increasing the slice gap from 0.1 cm to 0.2 cm increased the CE by 2% from 2.176% to 4.145%. Moreover, the same effect of the slice gap was observed with different grid sizes when estimating the volume of the DLPFC. Increasing only the grid size had a slight effect on the CE, and increasing the grid size from 1 × 1 pixels to 5 × 5 pixels resulted in an increase in the CE by less than 0.6%; however, less than 5% CE was achieved when using grid sizes of 2, 3, 4, and 5 pixels and 0.1 and 0.2 cm slice gaps. The effect of slice gapping on the CE was primarily observed when estimating the DMPFC volume, as increasing the gap from 0.1 cm to 0.2 cm raised the CE by 4%, whereas a similar increase was found when applying a 0.3-cm slice gap. For the DMPFC, <5% CE cannot be achieved unless a 0.1 cm slice gap is applied, regardless of grid size, but with a maximum of 5 × 5 pixels. A high level of precision was achieved when estimating the orbito-medial PFC by applying a grid size of 5 × 5 pixels and a 0.3 cm slice gap, which resulted in a 2.98% CE. On the other hand, using a slice gap >0.1 cm was not possible for all grid sizes, as applying a 0.2 cm slice gap resulted in > 5% CE; however, a 5 × 5 grid size with a 0.1-cm slice gap is considered a good stereological parameter because the CE was 2.986%.

The present study had several limitations that should be acknowledged. First, the use of a 2D T1-weighted TSE sequence, while compatible with stereological estimation, does not offer the isotropic resolution of 3D volumetric sequences. Although the Cavalieri method provides mathematically unbiased volume estimates with 2D data, future studies should incorporate 3D acquisitions to enhance spatial resolution and enable more comprehensive anatomical analysis. Second, no external gold standard or comparative method (e.g., automated segmentation or histological validation) was available to confirm the accuracy of our volumetric estimates; thus, our findings rely on internal consistency as evidenced by the high intra- and inter-rater reliability. Third, the volumetric analysis was limited to the right hemisphere, which may not fully capture the potential asymmetries in prefrontal development. Finally, the small sample size, while acceptable for a methodological pilot study, limits generalizability and should be expanded in future studies to ensure broader applicability.

## Conclusion

In the present study, we successfully developed and validated a novel methodology for the volumetric estimation of prefrontal cortical subfields in very pre-term infants using a combination of MRI and stereology. T1-weighted TSE images were acquired using a 1.5T scanner with the following parameters: repetition time (TR) = 3,023 ms, echo time (TE) = 150 ms, slice thickness = 3 mm, in-plane resolution = 0.7 mm, and matrix size = 256 × 256. Volume estimation was achieved using the Cavalieri principle combined with point counting, and applying optimized stereological parameters tailored to each subfield to achieve a CE below 5%. This method represents a significant advancement in neonatal neuroimaging, offering a precise, reproducible, and non-invasive method suitable for longitudinal monitoring. It may improve the early detection of neurodevelopmental risk and guide targeted interventions in preterm populations.
